# Same-Patient, Same–Time Point Evidence for Radiomics Benchmarking

**DOI:** 10.2196/110058

**Published:** 2026-10-06

**Authors:** Wenze Kan, Haoliang Pei, Mingxin Zhang

**Affiliations:** 1 School of Clinical Medicine, Jiamusi University, Jiamusi, China; 2 Department of Radiology, The First Affiliated Hospital of Jiamusi University, Jiamusi, China; 3 Department of Radiology, Zaoyang First People's Hospital, 183 Zhongxing Avenue, Zaoyang, Hubei, 441200, China, 86 17346379074

**Keywords:** radiomics, non–small cell lung carcinoma, neoadjuvant therapy, pathological response, meta-analysis, NSCLC

Jiang et al [1] provide a timely synthesis of studies on radiomics-based AI for predicting a pathological response after neoadjuvant immunochemotherapy in resectable non–small cell lung cancer. The pooled discrimination is encouraging. However, the comparison architecture and clinical estimand support a narrower conclusion than comparative superiority or surgery-modifying utility.

For pathological complete response (pCR), Table S11 in their paper [1] contrasts 16 AI validation datasets (n=1031 patients) with 2 conventional-criteria datasets (reported sample size: n=72). Table S7, however, reports PERCIST (Positron Emission Tomography Response Criteria in Solid Tumors) and RECIST 1.1 (Response Evaluation Criteria in Solid Tumors version 1.1) results in the same Deng et al [2] cohort, each totaling 36 participants; the corresponding AI entry in Table S5 is the separate 8-patient external-validation cohort [1]. Thus, the reported denominator sums 2 correlated test assessments rather than 72 unique participants and compares them with unmatched AI cohorts; this does not estimate relative accuracy [3]. The direction is also a trade-off: AI sensitivity was higher (0.77 vs 0.42), whereas specificity was lower (0.79 vs 0.97). The reported *Z* tests therefore cannot establish overall superiority without same-patient data and prespecified costs for false-positive and false-negative decisions.

Clinical use is further constrained by the target population. Eligibility required neoadjuvant therapy followed by radical resection, while included models used pretreatment, during-treatment, or pre-to-posttreatment imaging. The meta-analysis therefore conditions the pathological reference standard on reaching surgery and pools predictions made at different decision points. Patients who did not reach surgery because of progression, toxicity, or unresectability—the group most relevant to early redirection—are absent. In the CheckMate 816 trial [4], by contrast, pCR required no viable tumor in both lung and sampled nodes, and patients without surgery were counted as nonresponders in the intention-to-treat analysis. The pooled 68% positive posttest probability therefore does not establish readiness to guide surgical timing or organ preservation.

Two clinical examples also require correction. First, a 25% diameter decrease is stable disease under RECIST 1.1, not a partial response, which requires at least 30% [5]. Second, if pCR or a major pathological response is the positive condition, fibrosis mistaken for residual tumor creates a false negative, not a false positive.

A useful next step would be a treatment-initiation cohort with a locked model, fixed imaging time and threshold, explicit primary-tumor and nodal labels, and failure to reach surgery included in the estimand. AI, RECIST, and PERCIST should be compared in the same patients with dependence preserved, then assessed for calibration and net benefit at explicit surgical thresholds [6]. Such a design would test whether radiomics improves decisions, while the present synthesis more appropriately supports promising pathological-response prediction among patients who reach resection.

## Abbreviations

pCR: pathological complete response
PERCIST: Positron Emission Tomography Response Criteria in Solid Tumors
RECIST: Response Evaluation Criteria in Solid Tumors
